# Pulsatilla

**Published:** 1891-07-04

**Authors:** 


### PULSATILLA.

The tincture of Anemone Pulsatilla and Anemone Pratensis has been much vaunted of late as a specific for dysmenorrhœa. A good description of the plant is given in the U.S. *Dispensatory*, which describes the Anemone Pulsatilla as having a flower stalk from six to eight inches in height, with large, solitary flowers, having six dull violet-purple sepals, which are very downy on the exterior, and an involucre, which is at first near the flower and afterwards becomes more remote. The radicle leaves are on long stalks, and two or three times divided into long linear segments. It is a very common plant in England and Northern Europe. Anemone Pratensis, which affects Southern Europe, is especially distinguished by its having the involucre so close to the flower as to assume the appearance of a calyx. The herb contains a peculiar crystallisable principle, named anemonin, convertible into anemonic acid by the action of alkalies. Anemonin is deposited by water distilled from the fresh herb upon standing, and resembles camphor.

As regards its medical properties and uses, the same authority states that we have exact knowledge. It has been employed in Germany and other parts of Europe for the relief of amenorrhœa and dysmenorrhœa, and for other purposes. "In one or two trials very carefully made in nervous dysmenorrhœa, it has failed in our hands absolutely." The writer further refers to Baron Störck's opinion that Anemone Pratensis had proved useful in amaurosis and other complaints of the eye, in secondary syphilis, and in cutaneous diseases. Pulsatilla is supposed also to possess emmenagogue properties, and to exert an alterative influence over the mucous membranes, rendering it useful in ophthalmia, in catarrhal inflammation of the nostrils, throat, and respiratory passages. Dr. Peters, of New York, states that in large doses pulsatilla causes profuse perspiration, which may be followed by an eruption of small vesicles, or even pustules on the skin, and believes that therapeutically it is nearly equivalent to senega. M. Bovet has employed an alcoholic extract of pulsatilla in cases of dysmenorrhœa and ovaralgia with some success, this preparation being, in his experience, more active than the active principle anemonin. We believe that an indiscriminate employment of pulsatilla in such cases will prove eminently disappointing; in certain cases it is remarkably efficient. It has been commended for reducing the inflammation in metastatic orchitis, often subduing the pain and swelling in a very short time.

